# Monoplane Simpson’s Method Is Reliable for Left Atrial Volume Assessment in Small Dogs with Myxomatous Mitral Valve Disease

**DOI:** 10.3390/vetsci12100994

**Published:** 2025-10-15

**Authors:** Minsuk Kim, Minwoong Seo, Chul Park

**Affiliations:** 1 Department of Veterinary Internal Medicine, College of Veterinary Medicine, Jeonbuk National University, Iksan 54596, Jeonbuk, Republic of Korea; justin1866@jbnu.ac.kr (M.K.); minung0204@gmail.com (M.S.); 2 Onforest Animal Hospital, Gangnam-gu, Seoul 06230, Republic of Korea

**Keywords:** left atrial volume, myxomatous mitral valve disease, echocardiography

## Abstract

**Simple Summary:**

Left atrial enlargement is a key sign of heart disease progression in dogs with myxomatous mitral valve disease. This study compared two echocardiographic techniques—the monoplane Simpson’s method, which uses a single image view, and the biplane area–length method—to measure left atrial volume in small-breed dogs. Both methods effectively distinguished dogs with cardiac remodeling, but the results were not interchangeable, and their cutoff values differed. The monoplane Simpson’s method is straightforward and practical for routine veterinary use. Consistently applying one method and using the given cutoff values can help veterinarians monitor disease and support treatment decisions in daily practice.

**Abstract:**

Left atrial enlargement is a key marker of disease progression and prognosis in dogs with myxomatous mitral valve disease. Echocardiographic assessment of left atrial volume provides a more comprehensive measure than linear dimensions, yet different two-dimensional methods may yield variable results. This study aimed to compare the monoplane Simpson’s method of discs and the biplane area–length method for estimating left atrial volume indexed to body weight in dogs across different stages of disease. Dogs were prospectively evaluated with transthoracic echocardiography, and left atrial volumes were calculated using both techniques. Both indices clearly distinguished dogs with enlarged atria from controls and stage B1 patients. However, the two methods were not interchangeable, regardless of atrial size, as demonstrated by the Bland–Altman analysis. In conclusion, both techniques are clinically useful for assessing left atrial remodeling, but because they are not interchangeable, clinicians should consistently use one method. The monoplane Simpson’s method may be particularly practical for routine clinical application due to its convenience.

## 1. Introduction

Myxomatous mitral valve disease (MMVD) is the most common acquired cardiac disease in dogs. The staging and treatment of MMVD are based on American College of Veterinary Internal Medicine (ACVIM) consensus guidelines and most veterinarians perform diagnosis and treatment according to these guidelines in clinical practice [1]. In the diagnosis and monitoring of canine MMVD, the currently utilized modalities include thoracic radiography and echocardiography. Among these, echocardiography provides a more detailed assessment of cardiac structure and function. Currently, the main parameters used by veterinarians in echocardiography are two-dimensional (2-D) measurements, such as the left-atrium-to-aorta (LA/Ao) ratio and the normalized left ventricular internal diameter at diastole (LVIDdN). While several studies have investigated left atrial (LA) volume using transthoracic echocardiography in both healthy dogs and those with MMVD, a gold standard has yet to be established [2,3,4,5,6,7]. In human medicine, as well as in veterinary medicine, there are multiple methods for measuring LA volume using 2-D echocardiography, and these methods are not interchangeable [2,3,8,9,10,11]. There have been studies in dogs that indirectly calculate the regurgitant volume through the mitral valve using transthoracic echocardiography instead of directly measuring LA volume in dogs with MMVD [12,13,14]. While these studies have shown significant results, the complexity of the calculation led to their impracticality in routine clinical practice. Since the LA is anatomically asymmetrical and confined within the thoracic cavity, it may enlarge asymmetrically, thus assessing LA size using only 2-D measurements may be inaccurate. Additionally, discrepancies between the LA/Ao ratio and LA volume indexed to body weight have been observed in dogs with LA enlargement [15]. In human medicine, it is currently recommended to include LA volume measurement, particularly when assessing LA enlargement [16,17]. However, currently, only 10% of veterinarians utilize volume measurements when assessing LA enlargement using echocardiography [18].

LA volume has been assessed using echocardiography, with reference ranges reported in normal dogs, although the measurements vary depending on the imaging view and method used [2,4,19]. For volume measurement to be routinely adopted in clinical practice, it should be easy and convenient to perform. However, the biplane method requires a long-axis two-chamber view, which is not commonly used in clinical settings [18]. To date, no studies have investigated left atrial (LA) volume using the monoplane Simpson’s method of discs (SMOD) in dogs with myxomatous mitral valve disease (MMVD), categorized according to ACVIM guidelines.

The aim of this study was to evaluate the diagnostic value of LA volume derived from monoplane SMOD in small dogs (<15 kg) with MMVD. Moreover, we aimed to establish a cutoff value of LA volume (mL) divided by body weight (kg) to differentiate dogs with cardiac remodeling (LA enlargement). We also sought to compare the LA volume derived by the monoplane Simpson’s method to LA volume derived by the biplane area–length method and assess whether these methods are interchangeable. In addition, because image quality of LA can vary depending on atrial size, we investigated whether the degree of agreement between methods differed according to LA size.

## 2. Materials and Methods

This was a prospective observational study approved by the Institutional Animal Care and Use Committee (IACUC) of Jeonbuk National University (Approval No. NON2024-179). The study was conducted from February 2024 to February 2025 and included client-owned dogs that presented either for routine health screening or for cardiac evaluation due to suspected or diagnosed MMVD. Informed consent was obtained from all dog owners prior to inclusion. All enrolled dogs underwent a complete physical examination, thoracic radiography, and echocardiography. The diagnosis of MMVD was confirmed by the presence of a left apical systolic murmur on auscultation, along with evidence of mitral valve degeneration on B-mode echocardiography and mitral regurgitation on color Doppler imaging. Asymptomatic dogs diagnosed with MMVD were staged according to the ACVIM consensus guidelines. Dogs were classified as stage B2 if they met all the following criteria: left apical systolic murmur of ≥3/6, vertebral heart size (VHS) ≥ 10.5, LA/Ao ratio ≥ 1.6, and LVIDdN ≥ 1.7. Dogs not meeting these criteria were classified as stage B1. Dogs were classified as stage C if they had clinical signs consistent with congestive heart failure (e.g., tachypnea), evidence of increased pulmonary opacity on thoracic radiographs, and a positive response to diuretic therapy. Dogs were excluded if they had any cardiovascular disease other than MMVD or any systemic disease severe enough to potentially affect the cardiovascular system.

**Figure 1:**
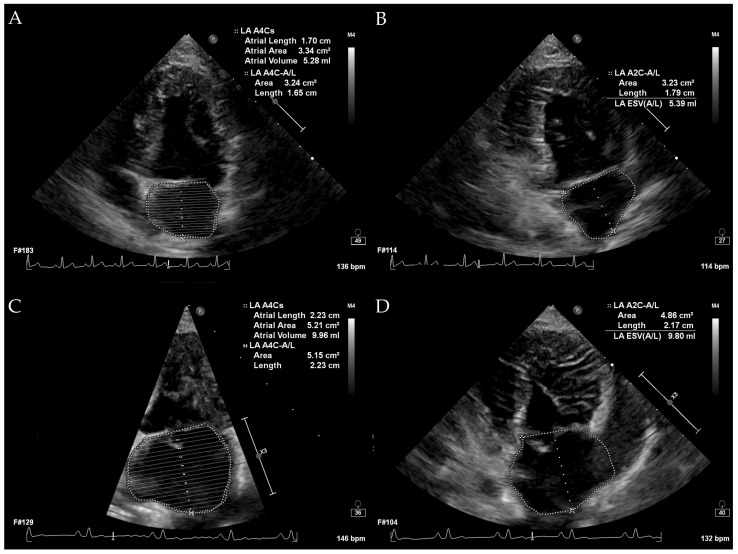
Echocardiographic measurements of left atrial volume. (**A**) illustrates the measurement in a normal dog using the left apical four-chamber view. (**B**) shows the measurement in a normal dog using the left apical two-chamber view. (**C**,**D**) depict measurements in a dog with LA enlargement using the left apical four-chamber view and left apical two-chamber view, respectively. In all images, the LA endocardial border was traced, carefully excluding the pulmonary veins and the left atrial appendage.

All echocardiographic examinations were performed using an EPIQ 7C ultrasound system (Philips, Bothell, WA, USA) equipped with a 3–8 MHz phased-array transducer by M.K and M.S. All procedures were conducted gently with manual restraint, and sedation with butorphanol was administered when necessary based on the patient’s condition. In cases of congestive pulmonary edema, diuretics were administered prior to echocardiography if clinically indicated. All echocardiograms were performed with continuous electrocardiography monitoring. Standard imaging planes included the right parasternal long-axis view, right parasternal short-axis view, left apical four-chamber view, and left apical two-chamber view. Additional views were obtained as needed. The 2-D, M-mode, pulsed-wave Doppler, continuous-wave Doppler, and color Doppler imaging were utilized according to the requirements of the examination. Normalized left anterior diameter (LADn), LA/Ao ratio, measured LVIDdN, and early diastolic transmitral flow (E peak) were measured from all dogs with both the measurement methods and scaling exponents based on previously published studies [4,20,21,22]. LA volumes measured by the monoplane SMOD method and the biplane area–length method were measured by tracing the endocardial border of the left atrium, carefully excluding the pulmonary vein and left atrial appendage, in left apical four-chamber view and left apical two-chamber view, one frame before mitral valve opening [19]. LA volume was calculated automatically by the built-in calculation formulas of the echocardiographic system. LA volume measurement is illustrated in Figure 1. LV volume was calculated using the monoplane SMOD in the left apical four-chamber view, one frame before mitral valve opening. All measurements were obtained as the average of three values, preferably from three consecutive cardiac cycles whenever possible.

A total of 64 dogs were reviewed in this study. The control group included 18 dogs (Mixed-breed: 9, Pomeranian: 2, Poodle: 2, Maltese: 1, Bichon Frise: 2). The stage B1 group included 26 dogs (Mixed-breed: 6, Maltese: 7, Poodle: 2, Chihuahua: 2, Miniature Schnauzer: 2, Cocker Spaniel: 1, Beagle: 1, Yorkshire Terrier: 1, Bichon Frise: 1, Pomeranian: 1, Pekingese: 1, Dachshund: 1). The stage B2 group included 8 dogs (Maltese: 3, Mixed-breed: 2, Poodle: 2, Chihuahua: 1). The stage C group included 12 dogs (Maltese: 6, Pomeranian: 3, Chihuahua: 2, Shih Tzu: 1). Table 1 summarizes the age, body weight, and sex of all dogs included in this study, categorized based on the criteria mentioned above. Ages of the control group were significantly lower than those in stages B1, B2, and C. Males were overrepresented in stages B2 and C. At the time of data collection, 3 out of 8 dogs (38%) in the stage B2 group were receiving pimobendan. In the stage C group, 7 out of 12 dogs (58%) were receiving pimobendan, 2 (17%) were receiving enalapril, 2 (17%) were receiving spironolactone, 1 (8%) was receiving amlodipine, and 6 (50%) were receiving furosemide.

Normality of the data was assessed using the Shapiro–Wilk and Kolmogorov–Smirnov tests. Based on the results, appropriate statistical methods were selected. All measured continuous variables were analyzed using the Kruskal–Wallis test. Pairwise comparisons among ACVIM stages for each index were performed using the Dunn–Bonferroni post hoc test. Receiver operating characteristic (ROC) curve analysis was conducted to evaluate the ability of echocardiographic parameters to distinguish between stages B1 and B2, as well as between control + B1 and B2 + C groups. Cutoff values, sensitivity, and specificity were determined using the Youden index. To assess the agreement between two LA volume indices—LA volume (mL)/body weight (kg) (LAvol/BW) measured by the biplane area–length method (LAvol/BW-A) and the monoplane Simpson’s method (LAvol/BW-S)—Bland–Altman analysis and proportional bias evaluation were performed. This analysis also aimed to determine whether the level of agreement varied depending on the presence of LA enlargement. A *p*-value of <0.05 was considered statistically significant.

## 3. Results

**Figure 2:**
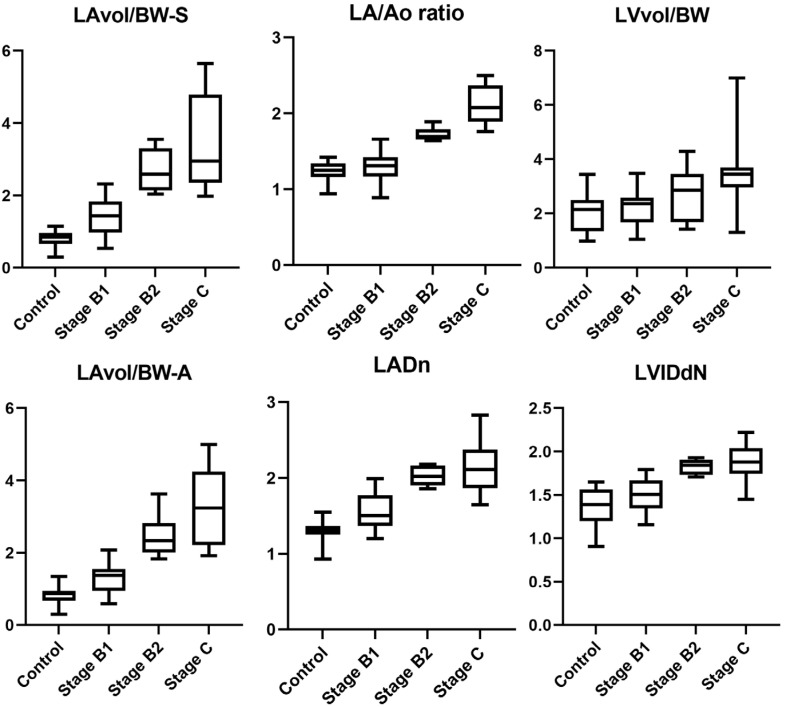
Box-and-whisker plots of echocardiographic measurements obtained in this study. Unlike other parameters, LVvol/BW showed no differences across ACVIM stages. When LAvol/BW-S and LAvol/BW-A were compared with conventional indices such as LA/Ao ratio and LADn, the volumetric indices demonstrated a broader spectrum of values, reflecting their ability to reflect a wider range of LA size.

All echocardiographic measurements obtained in this study are summarized in Table 2. Pairwise comparisons between ACVIM stages were conducted for each parameter. For LADn, significant differences were observed between the control group and stage B1, control and B2, control and C, B1 and B2, and B1 and C. For the LA/Ao ratio, significant differences were found between control and B2, control and C, B1 and B2, and B1 and C. For LVIDdN, significant differences were observed between the control group and stage B2, control and stage C, as well as between stages B1 and B2 and stages B1 and C. For E peak, significant differences were noted between control and B2, control and C, B1 and B2, and B1 and C. For LAvol/BW-A, significant differences were found between control and B2, control and C, B1 and B2, and B1 and C. For LAvol/BW-S, significant differences were identified between control and B1, control and B2, control and C, B1 and B2, and B1 and C. In contrast, for LVvol/BW, there were no significant differences among the different stages. Figure 2 shows the box plots of echocardiographic measurements measured in this study.

Receiver operating characteristic (ROC) analysis was performed to evaluate the diagnostic performance of echocardiographic volume indices in distinguishing between stage B1 and B2 dogs. The area under the curve (AUC) for LAvol/BW-A was 0.985 (95% CI: 0.953–1.017), and for LAvol/BW-S it was 0.955 (95% CI: 0.889–1.021), indicating excellent discriminatory power. In contrast, LVvol/BW showed poor performance with an AUC of 0.585, suggesting it was not useful for differentiating between stages B1 and B2. Using the Youden index, the optimal cutoff value for LAvol/BW-A was determined to be 1.83 (sensitivity 100%, specificity 92%), and for LAvol/BW-S it was 2.01 (sensitivity 100%, specificity 88%). Additionally, ROC analysis was conducted to distinguish between non-remodeled (control + stage B1) and remodeled (stage B2 + C) groups. The AUC for LAvol/BW-A was 0.994 (95% CI: 0.982–1.006), and for LAvol/BW-S it was 0.985 (95% CI: 0.962–1.007), again demonstrating high diagnostic accuracy. LVvol/BW remained non-discriminatory in this context, with an AUC of 0.639. The optimal cutoff values based on the Youden index were 1.83 for LAvol/BW-A (sensitivity 100%, specificity 95%) and 1.92 for LAvol/BW-S (sensitivity 100%, specificity 91%). These results are summarized in Table 3.

To assess the interchangeability between LAvol/BW-A and LAvol/BW-S, Bland–Altman analysis and proportional bias evaluation were performed. Additionally, the analysis was conducted separately for the non-remodeled group (control and stage B1) and the remodeled group (stage B2 and C) to investigate whether the degree of agreement varied depending on the extent of LA enlargement. C In the overall population, the mean difference between LAvol/BW-S and LAvol/BW-A was 0.11, with a standard deviation (SD) of 0.28. The limits of agreement (LOAs) ranged from −0.44 to 0.66. In the non-remodeled group, the mean difference was 0.28 (SD: 0.72), with LOA from −1.13 to 1.69. In the remodeled group, the mean difference was 0.22 (SD: 0.28), with LOA from −0.60 to 1.04. These results suggest a closer agreement between the two methods in dogs with LA enlargement (remodeled group) compared to those without remodeling. (Table 4).

## 4. Discussion

This study investigated LA volume indexed to body weight, measured using the monoplane SMOD method and the biplane area–length method, in small-breed dogs with MMVD, categorized according to the ACVIM consensus guidelines. We also investigated whether LAvol/BW-S and LAvol/BW-A differed across ACVIM stages, particularly in distinguishing between stages B1 and B2, as well as between the non-remodeled and remodeled groups. Furthermore, we evaluated whether LAvol/BW-S and LAvol/BW-A could be considered interchangeable.

In this study, conventional indices, as well as LA and LV volumes measured with echocardiography, were analyzed. Most variables showed significant differences between the remodeled and non-remodeled groups; however, LVvol/BW did not differ between groups and demonstrated no diagnostic utility. As MMVD progresses, increased preload induces LV eccentric hypertrophy and enlargement, accompanied by increases in LVIDdN, a finding that has also been reported in several previous studies [1,12,23,24]. The discrepancy between LVIDdN, which showed significant differences, and LVvol/BW, which did not, may be attributable to the pattern of LV eccentric hypertrophy in MMVD. LV enlargement in MMVD typically occurs in a transverse rather than longitudinal direction, with the mid-ventricular section being most prominently affected [25]. As a result, LVIDdN, which reflects enlargement of LV mid-section, shows significant differences, whereas LVvol/BW, which represents the overall chamber volume, may not demonstrate significant changes, although a trend toward increased LVvol/BW was observed without reaching statistical significance.

In this study, LA volume indices showed comparable performance to conventional echocardiographic parameters in differentiating ACVIM stages of MMVD. Previous studies suggested that volumetric measurements may be superior in detecting mild LA enlargement [7,15]. In the present study, when LA enlargement was defined as LA/Ao > 1.6 or LAvol/BW-S > 1.92, discrepancies were identified in only three cases. As no gold standard method for assessing LA size was available, it is difficult to conclude the superiority of one parameter over the other; rather, both appear to have similar ability in detecting LA enlargement. Although it remains unclear which parameter is definitively superior, as in human cardiology where both volumetric assessment and 2-D measurements of the LA are recommended, using both indices in combination may provide complementary value in clinical evaluation [26].

In this study, left atrial volume was measured using two echocardiographic methods: the monoplane SMOD and the biplane area–length method. The primary objective was to determine whether the monoplane SMOD, requiring only a single view (left apical four-chamber view), which is routinely acquired, could provide clinically meaningful values in small dogs with myxomatous mitral valve disease [18]. The biplane area–length method was included for comparison, as it is more precise but less practical in routine practice. We also hypothesized that larger LA, being easier to visualize, might improve the agreement between methods and potentially allow them to be interchangeable. Both techniques successfully identified LA enlargement across disease stages. However, the Bland–Altman analysis showed that the two methods were not interchangeable, and this finding was consistent regardless of LA size. Even when the LA was enlarged and better visualized, the monoplane method could not fully capture asymmetric chamber geometry, which likely contributed to the lack of interchangeability. Nevertheless, because Simpson’s method reliably distinguished dogs with left atrial enlargement, it represents a practical and clinically useful tool for routine assessment.

Both LA volume indices were able to distinguish between dogs with and without LA enlargement, which was further supported by ROC analysis. As summarized in Table 3, the proposed cutoff values provide clinically applicable thresholds that may assist veterinarians in identifying dogs with cardiac remodeling in practice. However, these values are derived from a limited sample size and should be validated in larger cohorts. Notably, in dogs with marked enlargement, the trends were inconsistent: in stage B2, LA volume measured by the monoplane Simpson’s method was larger, whereas in stage C, values obtained by the biplane area–length method were larger. This discrepancy can be explained by differences in the calculation principles. The monoplane SMOD estimates LA volume by integrating the endocardial contour of a left apical four-chamber view plane as a stack of cylindrical disks. When LA enlarges asymmetrically, as commonly seen in advanced remodeling (stage C), subvolumes may be excluded from the reconstruction, resulting in underestimation compared with the biplane area–length method. Conversely, in stage B2 where enlargement is relatively symmetric, the single left apical four-chamber view may capture the maximal cross-section, leading to relatively larger values by the monoplane SMOD.

This study has several limitations. It was observational in nature; although apical two-chamber views were specifically obtained for the purpose of the study, all other procedures were performed as part of routine clinical practice. Medication was not standardized, and both pimobendan and diuretics may have influenced LA size. Sedatives were also not controlled, although their effect on the measurements was likely minimal. Age was not uniformly matched across groups, and potential age-related influences could not be excluded. Male dogs were overrepresented in the later stages. In addition, left atrial volume indexed to body weight (LAvol/BW) may be inherently influenced by body condition score, and in advanced disease, progressive cachexia could lead to overestimation of atrial enlargement [27,28]. Furthermore, there was no established gold standard against which LA volume measurements could be validated, and the relatively small sample size limits the generalizability of the findings.

## 5. Conclusions

In this study, LAvol/BW effectively differentiated between dogs with and without LA enlargement using both the monoplane Simpson’s method and the biplane area–length method. However, the two methods were not interchangeable, indicating that clinical cutoff values for LAvol/BW may differ depending on the measurement technique. Among these, measuring LA volume by the monoplane Simpson’s method is simpler and more convenient for routine clinical practice. Therefore, the cutoff values established here can provide practical guidance, especially when using the monoplane method in veterinary practice.

## Figures and Tables

**Table 1:** Summary of characteristics for 64 dogs reviewed in this study.

	Control Group	ACVIM Stage B1	ACVIM Stage B2	ACVIM Stage C
Total (*n* = 64)	18	26	8	12
Age (years)	5.5 (3–7)	11.5 (7.8–14) ^a^	11.5 (8.25–14) ^a^	11 (10–13.75) ^a^
Weight (kg)	5.3 (3.67–7.49)	4.45 (3.34–6.86)	4.99 (2.89–6.83)	3.81 (2.94–4.62)
Sex (M:F)	10:8	17:9	6:2	9:3

Median (IQR) for continuous data. ^a^ Difference from control group. ACVIM—American College of Veterinary Internal Medicine, M—Male, F—Female.

**Table 2:** Measured echocardiographic variables of control dogs and dogs with MMVD included in this study.

	Control Group	ACVIM Stage B1	ACVIM Stage B2	ACVIM Stage C
LADn	1.315 (1.27–1.36)	1.51 (1.38–1.75) ^a^	2.02 (1.92–2.15) ^a,b^	2.11 (1.96–2.35) ^a,b^
LA/Ao ratio	1.25 (1.18–1.34)	1.31 (1.20–1.40)	1.70 (1.67–1.78) ^a,b^	2.08 (1.91–2.32) ^a,b^
LVIDdN	1.39 (1.21–1.56)	1.51 (1.36–1.65)	1.84 (1.76–1.90) ^a,b^	1.88 (1.76–2.02) ^a,b^
E peak	65.15 (60.53–74.83)	70.30 (62.50–78.00)	103.50 (92.28–110.75) ^a,b^	126.00 (98.75–131.00) ^a,b^
LAvol/BW-A	0.86 (0.71–0.94)	1.38 (0.96–1.55)	2.34 (2.09–2.82) ^a,b^	3.24 (2.39–4.22) ^a,b^
LAvol/BW-S	0.85 (0.68–0.95)	1.43 (1.02–1.81) ^a^	2.59 (2.16–3.01) ^a,b^	2.95 (2.40–4.57) ^a,b^
LVvol/BW	2.14 (1.54–2.43)	2.36 (1.72–2.55)	2.87 (1.98–3.17)	3.45 (2.97–3.60)

Median (IQR) for continuous data. ^a^ Difference from control group. ^b^ Difference from ACVIM stage B1. ACVIM—American College of Veterinary Internal Medicine, LADn—Normalized maximum left atrial dimension, LA/Ao ratio—Left-atrium-to-aorta ratio, LVIDdN—Normalized left ventricular internal dimension in diastole, E peak—Peak velocity of early diastolic transmitral flow, LAvol/BW-A—Left atrial volume (mL)/body weight (kg) measured by biplane area–length method, LAvol/BW-S—Left atrial volume (mL)/body weight (kg) measured by monoplane Simpson’s method, LVvol/BW—Left ventricle volume (mL)/body weight (kg).

**Table 3:** Summarization of ROC analysis of volumetric echocardiographic measurements used in this study.

Comparison	Index	AUC (95% CI)	Cutoff	Sensitivity	Specificity
B1 vs. B2	LAvol/BW-A	0.985 (0.953–1.017)	1.83	100%	92%
B1 vs. B2	LAvol/BW-S	0.955 (0.889–1.021)	2.01	100%	88%
B1 vs. B2	LVvol/BW	0.585	-	-	-
Non-remodeled vs. Remodeled	LAvol/BW-A	0.994 (0.982–1.006)	1.83	100%	95%
Non-remodeled vs. Remodeled	LAvol/BW-S	0.985 (0.962–1.007)	1.92	100%	91%
Non-remodeled vs. Remodeled	LVvol/BW	0.639	-	-	-

ROC—Receiver operating characteristic, AUC—Area under the curve, LAvol/BW-A—Left atrial volume (mL)/body weight (kg) measured by biplane area–length method, LAvol/BW-S—Left atrial volume (mL)/body weight (kg) measured by monoplane Simpson’s method, LVvol/BW—Left ventricle volume (mL)/body weight (kg).

**Table 4:** Bland–Altman analysis of agreement between left atrial volume indexed to body weight (LAvol/BW) measured by the monoplane Simpson’s method and the biplane area–length method in dogs with myxomatous mitral valve disease.

	Mean Difference	SD	Lower LOA	Upper LOA
Overall population	0.11	0.28	−0.44	0.66
Control + B1	0.28	0.72	−1.13	1.69
B2 + C	0.22	0.28	−0.6	1.04

SD—Standard deviation, LOA—Limit of agreement.

## Data Availability

The data presented in this study are available on request from the corresponding author. The data are not publicly available due to privacy/ethical restrictions.

## Abbreviations

The following abbreviations are used in this manuscript:

MMVD	Myxomatous mitral valve disease
ACVIM	American College of Veterinary Internal Medicine
2-D	Two-dimensional
LA/Ao	Left-atrium-to-aorta ratio
LVIDdN	Normalized left ventricular internal dimension in diastole
LA	Left atrium
SMOD	Simpson’s method of discs
E peak	Peak velocity of early diastolic transmitral flow
LADn	Normalized maximum left atrial dimension
ROC	Receiver operating characteristic
AUC	Area under the curve
LAvol/BW-S	Left atrial volume (mL)/body weight (kg) measured by the monoplane Simpson’s method
LAvol/BW-A	Left atrial volume (mL)/body weight (kg) measured by the biplane area–length method
LVvol/BW	Left ventricular volume (mL)/body weight (kg)
